# Comparison of Dynamic Contour Tonometry and Non-contact Tonometry in Older Patients Presenting with Headache or Vision Loss

**DOI:** 10.2174/1874364101812010104

**Published:** 2018-06-22

**Authors:** Edsel Ing, Angela Zhang, Evan Michaelov, Wendy Wang

**Affiliations:** 1 Department of Ophthalmology and Vision Sciences, University of Toronto, Toronto, Canada; 2 Schulich School of Medicine, Western University, Ontario, Canada

**Keywords:** Dynamic contour tonometry, Non-contact tonometry, Intraocular pressure, Agreement, Bland-Altman plot, Headache, Giant cell arteritis

## Abstract

**Background:**

Dynamic Contour Tonometry (DCT) is touted to be the most accurate tonometer for Intraocular Pressure (IOP) measurement. Non-Contact “air puff” Tonometry (NCT) may be the most commonly used tonometer for screening of IOP. Elevated IOP is important to exclude in patients presenting with headache or vision loss.

**Objective:**

To determine the agreement between DCT and NCT.

**Methods:**

The IOP of adult patients 50 years of age or older presenting with headache or vision loss for possible temporal artery biopsy were prospectively recorded. NCT and DCT measurements were obtained within thirty minutes. The right eye IOP measurements were compared with paired t-test, and Bland- Altman plot analysis. The left eye IOP measurements were subsequently analyzed for confirmation of results.

**Results:**

There were 106 subjects with complete right eye data, and 104 subjects with complete left eye data. The average age was 72 years, and 70% were female. The NCT IOP was on average 3.9 mm Hg lower in the right eye, and 3.5 mm Hg lower in the left eye compared with DCT. (p<.001) In the right eye the Bland-Altman analysis showed the 95% agreement interval between the two tonometers was -2.5 to 10.4 mmHg and in the left eye -3.0 to 9.9 mmHg.

**Conclusion:**

The IOP from NCT and DCT should not be used interchangeably because their level of disagreement includes clinically important discrepancies of up to 10 mm Hg.

## 1 INTRODUCTION

The role of Goldmann Applanation Tonometry (GAT) as the “gold standard” for Intraocular Pressure (IOP) measurement is being challenged given advances in technology such as the Pascal Dynamic Contour Tonometry (DCT) [1-3]. The DCT is more difficult to perform than GAT, but has been touted as one of the most accurate tonometers to date, as it is least affected by corneal thickness or other ocular biomechanical properties [3 - 5]. The DCT compares favorably with GAT with regards to repeatability [3]. Non-contact “air puff” tonometers (NCT) are being increasingly utilized for glaucoma screening [6] and convenient because they do not require anesthesia or sterilization. Only a handful of studies have compared DCT and NCT [7-10], predominantly in healthy patients. (Table **1**) We compare the results of DCT and NCT for elderly patients presenting with headache or vision loss.

## 2 MATERIALS AND METHODS

This study was approved by the Michael Garron Hospital IRB and compliant with the Declaration of Helsinki. Adult patients 50 years of age or older presenting with headache or vision loss, for consideration of temporal artery biopsy were recruited between March 2015 and April 2017. Patients who did not wish to participate in the study, and those who could not be positioned in the tonometry headrest for at least 5 seconds were excluded. Intraocular pressure was measured as part of the routine ophthalmic exam, and to exclude glaucoma as a cause of headache. Informed consent was obtained from all subjects.

The tonometers used were the automated Nidek Tonoref II (Nidek Co. Ltd, Aichi Japan) Non-Contact Tonometer (NCT), and the 2015 Pascal Dynamic Contour Tonometer (Ziemer Ophthalmic Systems AG, Port, Switzerland) hereafter referred to as DCT. All the NCT and DCT measurements were performed within a half hour of each other on the same day, usually between 1 pm and 3 pm. The right eye intraocular pressure (IOP) was measured first, and the NCT readings were performed first. Three NCT readings were performed on each eye by an ophthalmic technician, and the IOP result was averaged. The DCT readings were all performed by the same ophthalmologist (EI) who was masked to the results of the NCT. DCT readings were repeated until a quality reading of 1 or 2 was obtained. If the quality reading was worse than 2, three measurements were averaged. Corneal pachymetry was not available in our oculoplastics subspecialty clinic.

To avoid statistical errors with inter-eye correlation, the IOP from NCT and DCT of the right eye were compared, and the left eye data was later analyzed to determine consistency. Paired t-tests were used to examine the average agreement or bias.

The normality of the difference between the NCT and DCT measurements (ΔT) in each eye was tested with Shapiro Wilk and QQ plots. Paired t-tests were performed and the Bland-Altman limits of agreement technique was used to compare the two tonometers [11]. The range of agreement was defined as mean bias +/- 1.96 standard deviations. *A priori* we felt a limit of agreement of 2.5 mm Hg to be acceptable, and this was used for the *post hoc* sample size calculation.

Since central corneal thickness measurements (CCT) were not obtained, Bland-Altman analysis was repeated excluding the subjects with refractive errors (spherical equivalent) outside of the 5^th^ and 95^th^ percentile interval, and excluding subjects with LASIK or corneal transplant. Refractive error was measured by the autorefractor incorporated in the Nidek Tonoref II.

Stata 14.2 (StataCorp LLC, College Station, Tx) was used for most of the statistical analysis. If ΔT was skewed, Bland Altman analysis using the median of the differences was performed using Analyse-It 4.91. (Analyse-it Software, Ltd, Leeds, UK). The statistical calculations are documented in the Statistical Appendix. A *post hoc* sample size calculation for the Bland Altman analysis was completed using MedCalc 18.2 (MedCalc, Ostend, Belgium) with alpha 0.05, beta 0.20, an expected difference of zero, the standard deviation derived from the right eye IOP data (SDR), and maximum difference of 2.5 + 1.96*SDR.

## 3 RESULTS

All eligible subjects gave informed consent to participate in the study. There were 117 patients of average age 72.2 (standard devaiation +/-10.7) years of age. Eighty-one (70%) of the subjects were female. One hundred and six subjects had complete data from the right eye data for tonometry comparison, and 104 subjects had complete left eye data for comparison. Despite repeated attempts, the intraocular pressure using NCT could not be obtained in the right eye of six subjects, and the left eye of five subjects due to lack of cooperation. The DCT intraocular pressure was not obtained from the right eye of seven subjects and the left eye of nine subjects. In most cases where the DCT could not be obtained, the patient could not hold still for more than 5 seconds to obtain an adequate quality measurement; two subjects had prominent nasal pterygia which hampered DCT measurements. There was only one subject where neither the NCT or DCT intraocular pressure was recorded, due to right eye enucleation. Biopsy-proven giant cell arteritis was noted in sixteen patients with complete right eye IOP data and fifteen patients with complete left eye IOP data. The final ophthalmic diagnosis in the subjects in our study group included: neovascular glaucoma in one subject, central retinal artery occlusion in one subject, three patients with arteritic ischemic optic neuropathy (one of which was posterior), and eleven subjects with non-arteritic ischemic optic neuropathy.

The mean IOP for each device and for each eye is shown in Table **2**. All of the statistical calculations are listed in the accompanying Statistical Appendix. The Shapiro Wilk test (p=0.200) and Q-Q plot for the difference between the right eye NCT and DCT were consistent with normal distribution. However, in the left eye, the difference in the intraocular pressure on NCT and DCT showed a non-normal distribution on Q-Q plot with a Shapiro Wilk test p <.001.

Paired t-tests showed a statistically significant difference between ΔT for both the right and left eyes. (p <.001). In the right eye, the NCT IOP was on average was 3.9 mmHg lower than DCT and 3.5 mmHg lower in the left eye. The Pearson correlation coefficient between the NCT and DCT was 0.85 OD and 0.66 OS, both with p<.001.

**Fig. (1):**
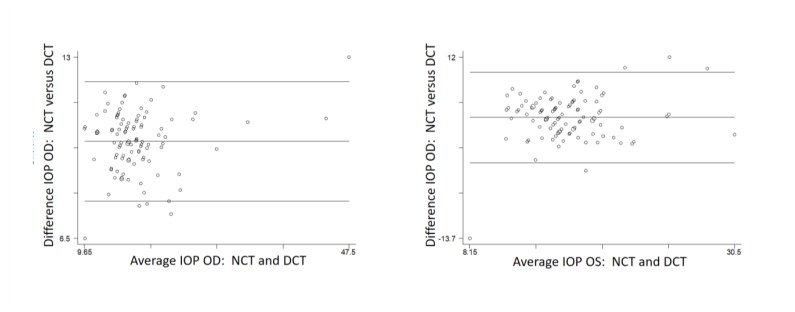
Bland-Altman plots comparing Dynamic Contour Tonometry *versus* Non-contact Tonometry.

On Bland-Altman analysis (Fig. **1**) the 95% limits of agreement between the two tonometers ranged from -2.5 to 10.4 in the right eye, and from -3.0 to 9.8 in the left eye (-3.2 to 10.4 on non-parametric analysis of the left eye). The two tonometers do not consistently provide similar measures because there is a level of disagreement that includes clinically important discrepancies of up to 10 mm Hg.

The *post hoc* sample size calculation for Bland Altman analysis was 58 pairs, and given our n >=104, this suggests the confidence intervals for the limits of agreement were not compromised. (see Statistical Appendix)

Subjects with spherical equivalent refractive errors beyond the 5^th^ and 95^th^ percentile for each eye were excluded, and the limits of agreement analysis repeated for the right eye and then the left eye. The analysis was also repeated for the higher myopes also. The refractive error subanalysis did not show any improvement in agreement between the tonometry measurements from either eye.

One subject had LASIK in the right eye, and two subjects had corneal surgery in the left eye. Repeat Bland-Altman plots excluding these subjects from the right then left eye analyses showed no change in the final result.

There was no statistically significant difference in the IOP between subjects with positive or negative temporal artery biopsy by NCT or DCT. Repeat Bland-Altman analysis excluding subjects with positive temporal artery biopsy did not improve the limits of agreement on tonometry measurements. The role of OPA in giant cell arteritis is discussed in a separate publication [12].

## 4 DISCUSSION

Goldmann Applanation Tonometry (GAT) has been in use for seven decades but its mire endpoints can be adversely affected by corneal biomechanics, and operator bias such that “a masked reader is required when IOP is a main outcome measure in study. One person physically applies the tonometer, while another reads the result” [1]. The role of GAT as the reference standard for IOP measurement measurement is being questioned given technological advances like the Pascal Dynamic Contour Tonometry (DCT) [1-3].

The DCT is more accurate than GAT because its digital readout removes operator bias, and because DCT measurements are not influenced by central corneal thickness, corneal curvature or anterior chamber depth [3-5]. However, DCT measurements require about 5 seconds of contact time with the cornea, and can be difficult to obtain. NCT is an increasingly utilized IOP-screening instrument [6] and can be performed by non-physicians without topical anesthetic, with little to no risk of cross-infection. As such it is valuable to know how DCT and NCT measure IOP relative to each other.

In this study of elderly patients presenting with headache or vision loss, fourteen had ischemic optic neuropathy. Our NCT read on average 3.7 mm Hg lower than the DCT. Other reports with younger patients, employing different tonometers, concluded that there was no agreement between NCT and DCT; on average the NCT measurement was 0.8 to 3.2 mm Hg lower than DCT, likely because NCT was more affected by central corneal thickness than DCT [7-10]

The lack of corneal pachymetry measurements is an acknowledged weakness of the study, but given the large difference in our NCT and DCT readings, even if CCT corrections were performed for the NCT, it is unlikely to alter the study conclusion. In fact, when Briceno et al adjusted their NCT measurements using Ehler’s algorithm for CCT, there was a *greater* difference between the DCT and “corrected” NCT measurements compared to the raw NCT [9]. Also, our analysis did not change with adjustments for higher refractive errors. Refractive error may be lenient, but inadequate proxy for CCT, as myopia has been correlated with thinner CCT [12-14]. Notwithstanding other sources have found no systematic correlation between CCT and refractive error [15].

## CONCLUSION

Our study of older patients presenting with headache or vision loss showed poor agreement between the IOP measurements from NCT and DCT. The intraocular pressures from these two devices should not be used interchangeably.

## Figures and Tables

**Table 1:** Studies comparing Dynamic Contour Tonometry and Non-contact Tonometry.

Author	Year	n	NCT	Mean Δ IOP (mm Hg)	LoA (mm Hg)	Mean Age (years)	Population
Burvenich	2005	294	Nidek NT 2000	2.7	?	?	Healthy
Erdurmus	2009	104	?	0.8	-0.8 to 3.0	61.4	Glaucoma or Ocular hypertension
Ito	2012	74	Topcon CT-70	3.2	-1.9 to 8.3	70.1	Non- glaucoma
Briceno	2016	45	Visionix VX 120	1.6	-2.9 to 6.1	47.2	Healthy
Present study (OD)	2017	106	Nidek Tonoref II	3.9	-2.5 to 10.4	72.2	Headache or Vision loss

Legend

N: number of patients.

NCT: non-contact tonometer.

DCT: dynamic contour tonometer.

Mean Δ IOP: mean difference in intraocular pressure from NCT versus DCT.

mm Hg = millimetres of mercury.

LoA: Limits of Agreement between DCT and NCT on Bland-Altman analysis.

OD: right eye.

**Table 2:** Mean Intraocular Pressure and Ocular Pulse Amplitude by Tonometry Type.

-	Right Eye n=106	Left Eye n=104
Mean NCT (mm Hg)	15.2 +/- 5.4	14.7 +/- 3.6
Mean DCT (mm Hg)	19.1 +/- 5.9	18.2 +/- 4.1
ΔT (mm Hg)	3.9 +/- 3.2	3.5 +/- 3.2
Paired t-test NCT versus DCT	p <.001	p <0.001
DCT OPA (mm Hg)	3.0 +/- 1.3	3.0+/- 1.3

Legend:

NCT = non-contact tonometer.

DCT = dynamic contour tonometer.

ΔT = mean difference in intraocular pressure (DCT – NCT) derived from individual measurements.

OPA = ocular pulse amplitude obtained from DCT.
